# Host Defense Peptides as Effector Molecules of the Innate Immune Response: A Sledgehammer for Drug Resistance?

**DOI:** 10.3390/ijms10093951

**Published:** 2009-09-09

**Authors:** Lars Steinstraesser, Ursula M. Kraneburg, Tobias Hirsch, Marco Kesting, Hans-Ulrich Steinau, Frank Jacobsen, Sammy Al-Benna

**Affiliations:** 1 Department of Plastic and Reconstructive Surgery, BG University Hospital Bergmannsheil, Ruhr University Bochum, Bochum, Nordrhein-Westfalen, Germany; E-Mails:u.kraneburg@yahoo.de (U.M.K.);tobias.hirsch@ruhr-uni-bochum.de (T.H.);hans-ulrich.steinau@bergmannsheil.de (H.-U.S.);frank.jacobsen@ruhr-uni-bochum.de (F.J.);sammy.al-benna@ruhr-uni-bochum.de (S.A.B.); 2 Department of Oral and Maxillofacial Surgery, Technische Universität München, Ismaninger Str. 22, München, Germany; E-Mail:marco.kesting@gmx.de (M.K.)

**Keywords:** host defense peptides, sepsis, innate immunity, antimicrobial peptides, bacterial infection, inflammation

## Abstract

Host defense peptides can modulate the innate immune response and boost infection-resolving immunity, while dampening potentially harmful pro-inflammatory (septic) responses. Both antimicrobial and/or immunomodulatory activities are an integral part of the process of innate immunity, which itself has many of the hallmarks of successful anti-infective therapies, namely rapid action and broad-spectrum antimicrobial activities. This gives these peptides the potential to become an entirely new therapeutic approach against bacterial infections. This review details the role and activities of these peptides, and examines their applicability as development candidates for use against bacterial infections.

## 1. Introduction

Throughout our history we have placed human health above our concern for the survival of pathogenic micro-organisms. We have sought the elimination of infectious diseases and the destruction of the micro-organisms that cause those diseases. Antibiotics and vaccines were one of the crown jewels of medical progress in the 20th century. At the start of the 21st century, we find ourselves in a peculiar and perplexing situation in that the pharmaceutical pipeline appears to have become less and less productive. Unfortunately, the initial promise of these miracle drugs was soon set back to some degree by the emergence of resistance to these agents among a broad variety of bacterial species and our ability to identify new agents to cope with the resistant bacteria is diminishing. Due to the threat of development of antimicrobial resistance as a result of misuse and/or overuse of antimicrobial agents, health care professionals should exercise caution and discretion when treating such infections with these agents [1].

In the 1940s hospitals in Europe and North America reported that bacterial infections were resistant to penicillin [2]. It took less than one year for resistance to penicillin to develop and interestingly the first description of penicillinase occurred 1 year before penicillin was ever used clinically [2]. Following this the first methicillin-resistant *Staphylococcus aureus* (MRSA) was identified in the 1960s [3]. From 1980–1986, there was a resurgence of infective diseases and mortality from these diseases in the United States of America increased by 64% to levels not seen since the 1940s [4]. The World Health Organization has stated that MRSA infections are now endemic worldwide. The rate of MRSA in Canadian hospitals increased from 0.46 to 5.90 per 1,000 admissions between 1995 and 2004 [5], which constituted an enormous increase in hospitalization and treatment costs, with the total cost per infected MRSA patient averaging $12,216 [6]. In 2002, the first clinical isolate of vancomycin-resistant *S. aureus* (VRSA) was identified [7].The struggle to gain the upper hand against infections continues to the present day. Medical advances against infectious disease have been hindered by changes in the patient population. Immunocompromised hosts now constitute a significant proportion of the seriously infected population, including patients that have been immunosuppressed by clinicians to prevent the rejection of transplants and to treat inflammatory diseases.

Over the past 50 years health care professionals and researchers have been fighting the increasing number of drug resistant bacteria, since such pathogens present significant clinical problems in managing infections that were easy treatable years before. Diseases once thought to have been nearly eradicated from the developed world, including tuberculosis, cholera and rheumatic fever, have risen again with renewed ferocity. These infectious diseases are major causes of morbidity and mortality worldwide. Therefore, a new paradigm in the treatment of infectious diseases is needed that is not compromised by the development of increasingly aggressive resistant strains of microbial pathogens and the rapid reduction of therapeutic efficacies of the available antibiotic therapies.

The increasing resistance of bacteria to conventional antibiotics has encouraged strong efforts to develop antimicrobial agents with new mechanisms of action. Perhaps research has now entered the post-antibiotic era, as there are currently major initiatives to develop new therapies, including host defense peptides (HDPs), which combine antimicrobial activity with angiogenic, immunomodulating and anti-inflammatory properties [8–10]. None of the clinically used antibiotics possess these features.

Equally important as their roles in the mediation of innate immune response are the roles that each type plays in recruiting B and T lymphocytes of the adaptive immune system to engage in specific antipathogen response. Their functions include antimicrobial, anti-endotoxic, anti-inflammatory activities and promoting wound healing. This aim of this review is to discuss the suitability of HDPs as potential therapeutic tools to treat infectious disease.

## 2. Cationic Host Defense Peptides

As an evolutionary co-development to prevent microbial colonization and tissue damage the production of HDPs of various structural classes have been isolated from a wide range of animal, plant, fungal and bacterial species [12]. As they have successfully retained their antimicrobial activity for millions of years, certain HDPs act as natural antibiotics, showing an exceptionally broad spectrum of activity, ranging from Gram-negative and Gram-positive bacteria to fungi and viruses [13–16]. It is conceivable that the relatively rapid development of microbial-resistance mechanisms may have evolved alongside the variety and high number of HDPs. At present, more than 1,220 HDPs are known, including over 940 HDPs in eurkaryotic organisms, listed in three databases [17–19].

According to their molecular composition, size, conformational structure, or predominant amino acid structure, HDPs can be divided into three main classes: linear α-helical structures without disulphide bonds (for example, cathelicidins, magainins and cecropins), β-sheet structures stabilized by characteristic disulfide bridges (for example, defensins), with predominance of one or more amino acids, and loop-structured peptides [13,20–22].

These latter two human classes constitute the less well-studied cathelicidins and defensins (Table 1) [23,24]. The α-helical structured hCAP-18, also known as LL-37, was first described in 1995 and is the only investigated antimicrobial peptide member of the cathelicidin family. Cathelicidins are a family of antimicrobial proteins found in most mammalian species. They consist of a highly conserved N-terminal domain, cathelin, and a variable C-terminal peptide, which is proteolytically released upon demand. It is mainly produced by leucocytes, epithelial cells and mucosal cells where it is stored in specific granules [25]. Its cationic C-terminal 37 amino acid domain, LL-37 displays broad antimicrobial activity mediated through direct interaction with and disruption of the microbial cell membrane. The enzyme responsible for cleavage of the proprotein in neutrophils is serine proteinase 3. In skin, the serine proteases kallikrein 5 and 7 were recently reported to mediate alternative processing of hCAP18 generating several novel peptide fragments, suggesting that peptide profiles may differ between tissues and biological conditions. This opens up a potential new area of research since their functional profile may differ. In addition to being antimicrobial, LL-37 is implicated in diverse biological processes, such as angiogenesis, chemotaxis, cytokine production, histamine release and wound healings [25–28,100]. Due to the simple linear structure of hCAP-18/LL-37 some bacteria are already able to inactive it by producing peptidases and proteases which degrades the peptide, for instance the V8 and aureolysin proteases from *S. aureus* and the outer-membrane protease PgtE from *Salmonella enterica* serovar typhimurium [29,30].

Defensins are a group of closely-related, Cys–Arg-rich, cationic HDPs comprised of 29 to 45 amino acid residues. The six conserved Cys residues that are a characteristic feature of defensins form three intramolecular disulfide bridges between the NH_2_- and COOH-terminal regions of the peptide, creating a cyclic, triple-stranded, amphiphilic β-sheet structure, making up the characteristic “defensin-like” fold and spatially separated hydrophobic and hydrophilic regions. These three intramolecular disulphide bridges stabilize its β-sheet structures and increase resistance to proteolysis, but also reduce the flexibility [31,32], although disulphide bridges are not necessarily essential for the antimicrobial activity of defensins [33]. Defensins can be divided into α- and β-defensins; the disulfide connectivities in α-defensins are Cys1–Cys6, Cys2–Cys4 and Cys3–Cys5 (the number indicates the location of the Cys residue in the amino acid sequence from the N-terminus), while in β-defensin they are Cys1–Cys5, Cys2–Cys4 and Cys3–Cys6 [34].

In human neutrophils, defensins comprise 30–50% of the granule proteins [35,36]. In human skin, defensins are produced mainly by keratinocytes, neutrophils, sebocytes or sweat glands [22] and are either expressed constitutively or after an inflammatory stimulus. α-Defensins secreted by neutrophils can be detected in biological fluids [37–40]. The concentration of α-defensins in human plasma under normal physiological conditions is about 40 ng/mL, as measured by ELISA [37]. This concentration increases 2- to 4-fold in patients with an inflammatory syndrome and reaches micromolar concentrations in septic patients [38]. In the plasma, α- and β- defensins bind unspecifically to high mass plasma proteins such as serum albumin, α2-macroglubulin and C1 complement, which decreases their anti-viral and anti-tumour activity [39,40]. Both α- and β- defensins have been found in human body fluids during inflammatory lung diseases, urinary tract infection and in tears after ocular surface surgery [22,41–46]. The relative structural stability of defensins has not caused their loss during the course of evolution and it can therefore be proposed that certain activities, such as the interaction with dedicated receptors, might have favored the development of more flexible peptides such as hCAP/LL-37. A better understanding of the function of hCAP/LL-37 and defensins in immunity has implications for the development of potential clinical therapeutics for the treatment of infection or cancer.

Recent studies have investigated the dichotomous role of HDPs in cancer biology. It has been shown that hCAP/LL-37 activates tumor cells resulting in increased cell growth *in vitro* and in an animal model [47,48]. In contrast, Bose *et al.* demonstrated that human beta defensin-1 (hBD1) induces rapid cytolysis of prostate cancer cells and that the PAX2 oncogene suppresses hBD1 expression in prostate cancer [49].

## 3. Activities of Host Defense Peptides

A microbial pathogen has the potential to enter any part of a host organism. Damaged skin can be a major portal of entry and allow multiplication of pathogens; tetanus and burn wound infections are clear examples. As mentioned previously, the innate immune system is the first line of defense as the adaptive immune response is not rapid enough to control the onset of infections. HDPs play a significant role in this process [14,15,50,51]. Their characteristics, including angiogenesis, anti-microbial, chemotaxis, cytokine production, histamine release, lipopolysaccharide (LPS)-binding properties and other immunomodulatory activities can lead to control infection and allow the appropriate activation of adaptive immune responses [52–54].

A correlation between the severity of the disease and the level of HDP production has been demonstrated in several studies. [55,56] Morrison *et al.* could demonstrate increasing susceptibility to infections caused by *S. aureus* in ß-defensin-2 knockout mice and isolated Dermcidin (DCD) peptide DCD-1L produced by eccrine sweat glands in the skin has been shown to stimulate the production of cytokines/chemokines by human keratinocytes [55,56]. Reduced expression of DCD in sweat of patients with atopic dermatitis has been associated with high susceptibility of these patients to skin infections and altered skin colonization [57]. In contrast, overexpression or exogenous application of HDPs have a protective effect in animals [51,58,59]. Clinically overexpresson can lead to increased protection against skin infections as seen in patients with psoriasis, rosacea and inflammatory skin-diseases such as acne vulgaris which is rarely associated with clinical superinfection [60]. The expression of many HDPs increases during infection and inflammation. For instance HBD-2 is upregulated in various cell types such as monocytes, epithelial cells and keratinocytes during bacterial infections and by stimulation from different bacterial components that activate the Toll-like receptor (TLR) to nuclear factor (NF)-kB pathway [61–63]. In addition, decreased defensin levels in burn injury may facilitate infection and subsequent sepsis [64].

Human skin is always in contact with the environment and is covered with a characteristic microflora of non pathogenic bacteria (commensals). HDPs are mainly associated with inflammatory lesions and wounds, but some are also focally expressed in skin, including β-defensins, RNase 7, S100-protein Psoriasin and hCAP-18/LL-37 in the absence of inflammation [22]. This may be due to an imbalance between impaired defense and increased susceptibility to infection [16].

Hancock *et al.* demonstrated that HDP antimicrobial activity was not necessarily required for protection *in vivo* and an HDP with no antimicrobial activity was found to be protective in animal models of *Staphylococcus aureus* and *Salmonella enterica* infection, implying that a host defense peptide can protect by exerting immunomodulatory properties [54]. HDPs have hitherto been considered for their antibiotic properties, however, some of them, particularly defensins, evolved to also exhort a large variety of other functions [54]. This raises the question as to what HDPs really are: are they principally antimicrobials or are they modulators of innate and adaptive immune responses, or both? It has been proposed that some HDPs, for example defensins, have traded their antibiotic capacities to acquire immunomodulatory functions presumably to reverse suppressive microbial functions and to elicit more robust host inflammatory and adaptive responses [54].

## 4. Strategies and Functional Properties of Host Defense Peptides

HDPs belong to the most rapid evolving group of mammalian peptides [65,66] and have developed broad-spectrum antimicrobial properties, such as direct killing of Gram-negative and Gram-positive bacteria, fungi, and parasites [12,67]. A therapeutic goal is to engineer peptides with high efficacy and target specificity. In order to achieve this goal, the mechanisms of actions of the HDPs must be understood.

There are distinctive different external and internal target mechanisms. One can distinguish between peptides that permeabilize and/or disrupt the bacterial cell membrane and peptides that translocate through the cell membrane and interact with a cytosolic target [68–70].

The antimicrobial activity of HDPs as membrane-agents, possessing a secondary α-helical peptide structure, depends on the presence of an ionic milieu that is comparable to the conditions found in mammalian body fluids [68–70]. The hydrophilic, cationic part is proposed to initiate electrostatic interaction with the negatively charged components of the membrane of microbes; the hydrophobic portion is supposed to permit the HDPs to insert into and permeate the membrane.

The positive charge provides some degree of selectivity towards negatively charged microbial cell envelopes and cytoplasmic membranes. These molecules tend to exhibit intrinsic specificity for microbial invaders and are relatively much less toxic for the metazoan host’s cells. This specificity endows the animal with an “innate” immunity, in contrast to the better studied acquired immunity conferred by the clonal expansion of B and T cells. The possible importance of this system as a check on infection is evident when one considers that most bacteria have generation times of 20–30 minutes whereas the mounting of a specific immune response, dependent on the growth of mammalian cells, may take days or weeks. Although many cationic peptides demonstrate direct antimicrobial activity against bacteria, fungi, eukaryotic parasites and/or viruses [71–73], it has also been established that many also have a key modulatory role in the innate immune response and present an important link between the innate and adaptive immune responses [74].

These HDPs penetrate the outer membrane of Gram-negative bacteria by self-promoted uptake due to hydrophobic or electrostatic interactions with lipid A or polyanionic surface LPS (LPS) [11,75]. Other mechanisms to kill the bacteria cell are possible due to transmembrane channels, which cause changes in permeability of the negatively charged phospholipid membrane. Other models such as barrel stave, lipid flip-flop and carpet models are currently discussed as potential ways for HDPs to penetrate bacterial membranes [76,77]. Furthermore, DNA and other intracellular targets can be affected, leading to cell destruction [77,78]. Many HDPs have additional properties such as anti-endotoxic and anti-inflammatory properties, promote apoptosis, influence cell differentiation, induce cytokine and chemokine release, stimulate mast cell degranulation, possess chemotactic properties and promote wound healing [79–82].

As participants in a competent immune response to invading bacteria, HDPs act as immune activators by induction of transcription and secretion of cytokines such as IL-8, IL-18, TNF-α, GM-CSF, IL-1ß [56,83,84], and induce histamine release from mast cells [85–87]. Certain bacteria are better at provoking an overwhelming immune response from the cells of the innate immune system, such as monocytes, macrophages, and dendritic cells [80,88], which connect the innate immune system with the adaptive immune system. A hyperinflammatory response exposes the organism to the pathological processes of sepsis and may lead to organ failure and death [89–91]. In this case, HDPs may operate as immune response suppressors to prevent a hyperinflammatory response producing severe tissue damage (self-damage) from an overwhelming immune response [23].

Endotoxin covers more than 90% of the outer monolayer of Gram-negative bacteria. For the microbe, endotoxin works as a protective barrier against antibiotics and for the host immune system it is an effector molecule, which is recognized by and activates the innate immune system. In some cases life-threatening complications occur caused by antibiotics stimulated Gram-negative bacteria endotoxin release [92–94]. The human antimicrobial peptide hCAP18/LL-37 is a multifunctional modulator of innate immune responses, its ability to neutralize endoxin inhibits the production of proinflammatory cytokines such as TNF-α and IL-6 [23,25]. Scott *et al*. demonstrated that structurally different cationic antimicrobial peptides block the interaction between LPS and LPS-binding protein, inhibiting transport and binding of LPS to CD14 receptor [95]. Cirioni *et al*. reported protective effects of hCAP18/LL-37 in lethal sepsis caused by Gram negative bacteria in experimental rat models of peritoneal sepsis. The human cathelicidin LL-37 proved to be similarly successful as three commonly used antibiotics in lowering lethality [96]. Furthermore, HDPs interact directly with eukaryotic cells [43] and can induce alterations in the transcription of hundreds of genes in cells of the innate immune system [97]. In addition cathelicidins and defensins promote cell proliferation and wound healing [98–102].

## 5. Current Limitations of Host Defense Peptides

As HDPs have a different mode of action compared to conventional antibiotics, they are less affected by multiresistant microbes. However certain HDPs have limitations and some bacteria have developed mechanisms to avoid direct killing by HDPs [103,104], through extracellular and intracellular resistance mechanisms, including reduction of negative surface charge of the cell wall [103,104] and efflux mechanisms [103]. HDPs with a simple linear or α-helical structure are susceptible to proteolysis and are targeted by several microbial proteases. Nevertheless, HDPs can be prevented from reaching the bacterial cell membrane by bacterial secreted proteins. Examples of bacterial secreted proteins include *Streptococcus pyogenes* with streptococcal inhibitors of complement (SIC) proteins, and *Staphylococcus aureus* with staphylokinase [105,106]. In addition, certain HDPs can be actively extruded from bacterial cells, for example by the MtrCDE multiple drug resistance exporter in both *Neisseria gonorrhoeae* and *Neisseria meningitidis* [107,108]. A potential concern that has been noted is that bacteria can also reduce the net anionic charge of their cell envelope, reducing the affinity of HDPs to them. However, these mechanisms are relatively inefficient compared to those that inhibit the effect of conventional antibiotics [109]. Furthermore, only direct killing activity is affected by resistance mechanisms of bacteria whereas immunomodulatory function is untouched.

A new paradigm for the treatment of microbial disease is through the modulation of innate immune responses. The importance of HDPs as antimicrobial agents versus their role as immunomodulators is somewhat controversial. In addition, the pretence of host defense is relative, as the cytotoxic activities of HDPs may have originally evolved to defend the producer bacteria from eukaryotic cell predation [110]. HDPs can trigger autoimmunity through immunomodulation. Autoimmunity and host antimicrobial immunity are inextricably linked, as effector responses that cause inflammatory tissue damage are the same ones that mediate effective host defence. Therefore, immunotherapeutic regimens that target common pathways of the immune system inevitably elicit both desirable and undesirable consequences. HDPs can also interact with human membranes and this is at least partly responsible for cytotoxicity of some peptides at high concentrations [69]. However, distinct composition of mammalian membranes, with preferential localisation of anionic phospholipids into the inner leaflet, offers some protection [69]. These cytotoxic functional properties of HDPs are generally are thought to be associated with their pore-forming activities as multimers in biological membranes leading to self-promoted uptake [111,112] a mechanism that has been further described by the Shia-Matsuzaki-Huang model [113–115]. It is also important to be aware that as HDP hydrophobicity increases, generally there is seen a corresponding increase in antimicrobial activity. However, once hydrophobicity exceeds a certain level, selectivity between prokaryotic and eukaryotic membranes is lost, with a concomitant increase in cytotoxicity [116]. It has been demonstrated in animal models of infection that low dose hCAP/LL-37 therapy gives a significant survival benefit over high dose and control groups. In both studies, high dose hCAP/LL-37 therapy was associated with a higher mortality rate when compared to both low dose and control groups [117,118]. LL-37 has been shown to have non-selective cell toxicity, hemolytic activity and causes DNA fragmentation in cell cultures [119,120]. Many other HDPs were shown to be cytotoxic [120]. These setbacks led to increased efforts to develop novel synthetic HDPs with less cytotoxic effects and improved antimicrobial activity.

The concentrations of HDPs found at many body sites (for example, the mucosa) are inconsistent with a primary role in direct antimicrobial (killing) action. In addition, HDPs show decreased activity at the physiological conditions found in humans due to their sensitivity to both saline solutions and monovalent/divalent ions such as Mg^2+^ or Ca^2+^ in serum [54,121,122]. Hancock *et al*. observed that beta defensin completely loses its activity in concentrations of 100 mM 0.9% NaCl [54]. The weak antimicrobial activity under physiologically relevant conditions has been observed with many HDPs and has led to increasing discussion as to whether the primary function of certain HDPs is to kill bacteria directly [123,124]. Studies have shown that at human physiological conditions HDPs have minimal direct antimicrobial activity but retain their immunomodulatory properties to provide *in vivo* protection [121,122].

## 6. Are We Entering the Post-Antibiotic Era?

Increased levels of HDPs are often found in inflamed or infected skin areas indicating a role of these peptides in the protection from infection. Several studies have indicated that HDPs have therapeutic potential as topical anti-infectives [73,123–126]. The broad spectrum of antimicrobial activity, the low incidence of bacterial resistance and their function as immunomodulatory agents are attractive features of HDPs for clinical use. Nevertheless, the morbidity of their cytotoxic effects on host cells may limit their use but inform the development of synthetic cationic peptides with diminished cytotoxic profiles and enchanced therapeutic properties, such as the ovispirin (sheep HDP) –derived designer peptide proline-novispirin G10, which provides broad-spectrum antimicrobial activity with low haemolytic and cytotoxic activities [73,125]. Removal of hydrophobic amino acids from the N-terminal end of hCAP18/LL-37 holds promise as a template for the reducing saline sensitivity but maintaining antimicrobial activity [128].

An alternative strategy is gene transfer and the development of target specific HDPs (STAMP = Specific target HDPs); they have already shown new ways to combat infection and inflammation with HDPs *in vitro* and *in vivo* [73,129,130]. Another promising approach is the combined application of two or more HDPs [121] as well as the combination of HDPs with commonly used antibiotics [132] in an effort to prevent the development of antimicrobial resistance and to provide the infected host with optimized antimicrobial therapy [131,132].

The clinical application of HDPs is still in its infancy. However, under the few available clinical studies [133] four peptides demonstrated clinical effectiveness [109]. In 2000 Levin *et al* showed decreased morbidity in children suffering from meningococcal sepsis, who were treated with the designer host defense peptide, rBPI21 (Neuprex), derived from bactericidal/permeability-increasing protein [BPI], a human host defense protein produced by polymorphonuclear leukocytes [134]. Currently, there are ongoing clinical trials investigating therapeutic effects of rBPI21 in burn patients and MSI-78 in diabetic foot ulcers and impetigo.

The bovine indolicin-derived Migenix MX-226 has already proceeded to a phase 3B study for the treatment and prevention of catheter-associated infections [133,109]. Promising candidates for the treatment of acne are the designer peptides XOMA 629 and MBI 594AN with bactericidal effects against Propionibacterium acnes [135]. This agent is expected to be available for clinical therapy in the near future.

## 7. Conclusions

The emergence of bacterial resistance to antibiotics has led to an increased urgency to explore alternative means of combating pathogenic assault. HDPs are rapidly emerging as attractive candidates for anti-microbial treatment. HDPs have proven to have an important role in host defenses, with many desirable features, and represent an entirely novel class of antimicrobials. The contrasting direct antimicrobial and immunomodulatory activities make these peptides valuable in the design of alternatively directed therapeutic agents and as tools in dissecting the variations in the mechanisms that underpin these diverse activities. In addition, their small size makes them potentially exciting prototypes for development as novel immunomodulatory drugs, especially since their collective ability to enhance chemokine production, induce chemotaxis, and block endotoxin responses. Future work will involve optimizing these HDPs and better characterizing their immunomodulatory properties to further illuminate their potential as novel therapeutic agents. In addition as HDPs act via a wide acting innate immune system rather than directly on microbe there should not be resistance. There is potential for the clinical success of antimicrobial HDPs. And although they do not solve all of the issues created by the problem of antibiotic resistance, they do offer a prospectively exciting new tool in the clinician’s armamentarium.

## Figures and Tables

**Table 1.** Functions of HDPs in combating infections and modulating the immune response.

**Host Defense Peptide**	**Site of Expression**	**Immunomodulatory Function**
***Human cathelicidin (cationic, α-helical structure, consist of a highly conserved N-terminal domain, cathelin, and a variable C-terminal peptide) [13, 16, 23, 25, 51, 53, 54, 75, 79, 95, 100]***		
hCAP18/LL37	Neutrophils, keratinocytes, epithelial cells of skin and testis, gastrointestinal and respiratory tract, mast cells, monocytes/macrophages, CD4+ cells, myelocytes, wound and blister fluid, cervix vagina, esophagus, mouth, tongue	Broad antimicrobial activity, antiviral and antifungal activity, endotoxin-binding properties, modulation of pro-inflammatory response, chemotactic, influence of cell proliferation and differentiation, promoting wound healing and angiogenesis, induction of gene expression, induction of adaptive immunity
***Human α-Defensins (human neutrophil proteins; closely-related to human cathelicidin, Cys–Arg-rich, cationic, disulfide bridges, β-sheet structure) [22, 36–40]***		
HNP-1 to -4	Azurophilic granules of neutrophil granulocytes, B-cells, natural killer cells, T-cells	Killing of phagocytosed microorganisms, antimicrobial activity against Gram-positive and gram-negative bacteria, antiviral (HSV, CMV, HIV-1) properties, exotoxin-inactivation, chemotactic for monocytes, T-cells, immature dendritic cells, upregulation of tumor-necrosis factor α (TNF-α) and IL-1, downregulation of complement activation, promotion of DC activation
HD-5 and -6	Paneth cells granules of neutrophils, natural killer cells	Microbicidal activity against *Escherichia coli*, *Staphylococcus aureus*, *Listeria monocytogenes, Salmonella typhimurium*, C. albicans, induction of IL-8
***Human-β-Defensins (closely-related to human cathelicidin, Cys–Arg-rich, cationic, disulfide bridges, β-sheet structure) [13, 34–36, 61, 105]***		
hBD-1	CD4+ and CD8+ T-cells, dendritic cells, epithelial cells of skin, respiratory, gastrointestinal and urogenital tract, trachea, uterus, pancreas, thymus, testis, vagina, gingival intestine, conjunctiva, cornea, lacrimal and buccal mucosa, tongue, salivary gland, mammary glands, limb joints, astrocytes, microglia	Broad antimicrobial activity, antiviral and antifungal activity, chemotactic, induction of chemokines and cytokines, recruiting immune cells, induction of adaptive immunity and pro-inflammatory cytokines such as IL-8, -18 and -20, degranulation of mast cells, promotion of phagocytosis, induction of dendritic cell maturations by TLR-4, LPS and LTS binding properties, inhibition of MMP-inhibitors (TIMP-1/-2)
hBD- 2	Mast cells, CD4+ and CD8+ T-cells, dendritic cells, skin, oral, pulmonl, gastric epithelia, conjuctiva, cornea, astrocytes	
hBD- 3	Monocytes, CD4+ T-cells, oral, respiratory tract, gastrointestinal tract, urinary and skin epithelial cells, uterus, placenta, testis, esophagus, heart, neutrophils, trachea, skeletal muscle, tongue, kidney, liver gastrointestinal tract, oropharynx, tonsils, salivary glands	

**Table 2.** Host defense deptides in commercial development.

**Drug**	**Stage of development**	**Medical use**
BL2060 (a synthetic compound comprising fatty acid and lysine copolymers)	Lead optimization	Anti-infective
CSA-13 (cationic steroid (ceragenin) that mimics host-defense peptides)	Preclinical	Anti-infective
CZEN-002 (synthetic 8-mer derived from - melanocyte–stimulating hormone)	Phase 2b	Vulvovaginal candidiasis
HB-50 (synthetic natural peptide mimetic of cecropin)	Preclinical	Anti-infective
HB-107 (19-amino-acid fragment of cecropin B)	Preclinical	Wound healing
hLF-1-11 (small peptide derived from human lactoferrin)	Phase 2	Allogeneic bone marrow stem cell transplantation–associated infections
IMX942 (5-amino-acid peptide)	Lead optimization	Immunomodulation; treatment of fevers and neutropenia in chemotherapy patients
MSI-78	Phase IIIb	Anti-infetive; Wound healing
Omiganan pentahydrocholoride/CP-226/MX-226/CLS001 (12-mer analog of bactolysin)	Phase 3b	Prevention of catheter-related infections; dermatology-related infections
MBI 594AN	Preclinical	Anti-infective
Mersacidin (bacteriocin)	Preclinical	Gram-positive infections
Plectasin (fungal defensin)	Preclinical	Systemic anti–Gram positive, especially pneumococcal and streptococcal infections
PAC113 (based on the active segment of histatin 5 protein found in human saliva)	Investigational New Drug (IND) approval	Oral candidiasis
PTX002 (33-mer peptide) PTX005 (12-mer peptide), PTX006 (N-acylated analog of PTX005) and PTX007 (a nonpeptidic structur analog of PTX005)	Discovery	Broad-spectrum antimicrobial antiendotoxin
Peptidomimetics (derived from the arylamide, calixarene, hydrazide and salicylamide series)	Discovery/preclinical	Anti-infectives; antimicrobial polymers and coating materials
rBPI21	Phase IIIb	Anti-infective; Allogeneic bone marrow stem cell transplantation–associated infections; Prevention of burn infections
XOMA 629	Phase 2a	Anti-infective

## References

[1] Davies J Origins and evolution of antibiotic resistance Microbiologia 1996 12 9 16 9019139

[2] Barber M Rozwadowska-Dowzenko M Infection by penicillin-resistant staphylococci Lancet 1948 2 641 644 18890505 10.1016/s0140-6736(48)92166-7

[3] Benner EJ Kayser FH Growing clinical significance of methcillin-resistant Staphylococcus aureus Lancet 1968 2 741 744 4175546 10.1016/s0140-6736(68)90947-1

[4] Armstrong GL Conn LA Pinner RW Trends in infectious disease mortality in the United States during the 20th century JAMA 1999 281 61 66 9892452 10.1001/jama.281.1.61

[5] Surveillance for methicillin-resistant Staphylococcus aureus in Canadian hospitals--a report update from the Canadian Nosocomial Infection Surveillance Program Can. Commun. Dis. Rep 2005 31 33 40 15693179

[6] Goetghebeur M Landry PA Han D Vicente C Methicillin-resistant Staphylococcus aureus: A public health issue with economic consequences Can. J. Infect. Dis. Med. Microbiol 2007 18 27 34 18923684 10.1155/2007/253947 PMC2542887

[7] Clark NC Weigel LM Patel JB Tenover FC Comparison of Tn1546-like elements in vancomycin-resistant Staphylococcus aureus isolates from Michigan and Pennsylvania Antimicrob. Agents. Chemother 2005 49 470 472 15616340 10.1128/AAC.49.1.470-472.2005 PMC538911

[8] Deans KJ Haley M Natanson C Eichacker PQ Minneci PC Novel therapies for sepsis: A review J. Trauma 2005 58 867 784 15824673 10.1097/01.ta.0000158244.69179.94

[9] Ziebuhr W Xiao K Coulibaly B Schwarz R Dandekar T Pharmacogenomic strategies against resistance development in microbial infections Pharmacogenomics 2004 5 361 379 15165173 10.1517/14622416.5.4.361

[10] Wesche-Soldato DE Swan RZ Chung CS Ayala A The apoptotic pathway as a therapeutic target in sepsis Curr. Drug Targets 2007 8 493 500 17430119 10.2174/138945007780362764 PMC1852483

[11] Tossi A Sandri L Giangaspero A Amphipathic, alpha-helical antimicrobial peptides Biopolymers 2000 55 4 30 10931439 10.1002/1097-0282(2000)55:1<4::AID-BIP30>3.0.CO;2-M

[12] Hancock RE Scott MG The role of antimicrobial peptides in animal defenses Proc. Natl. Acad. Sci. USA 2000 97 8856 8861 10922046 10.1073/pnas.97.16.8856 PMC34023

[13] Hancock RE Peptide antibiotics Lancet 1997 349 418 422 9033483 10.1016/S0140-6736(97)80051-7

[14] Zasloff M Antimicrobial peptides of multicellular organisms Nature 2002 415 389 395 11807545 10.1038/415389a

[15] Hancock RE Cationic peptides: Effectors in innate immunity and novel antimicrobials Lancet Infect. Dis 2004 1 156 164 11871492 10.1016/S1473-3099(01)00092-5

[16] Steinstraesser L Oezdogan Y Wang SC Steinau HU Host defense peptides in burns Burns 2004 30 619 627 15475133 10.1016/j.burns.2004.05.013

[17] Brahmachary M Krishnan SP Koh JL Khan AM Seah SH Tan TW Brusic V Bajic VB ANTIMIC: A database of antimicrobial sequences Nucleic Acids Res 2004 32 D586 D589 14681487 10.1093/nar/gkh032 PMC308766

[18] Fjell CD Hancock RE Cherkasov A AMPer: A database and an automated discovery tool for antimicrobial peptides Bioinformatics 2007 23 1148 1155 17341497 10.1093/bioinformatics/btm068

[19] Wang G Li X Wang Z APD2: The updated antimicrobial peptide database and its application in peptide design Nucleic Acids Res 2009 37 D933 D937 18957441 10.1093/nar/gkn823 PMC2686604

[20] Andreu D Rivas L Animal antimicrobial peptides: An overview Biopolymers 1998 47 415 433 10333735 10.1002/(SICI)1097-0282(1998)47:6<415::AID-BIP2>3.0.CO;2-D

[21] van’t Hof W Veerman EC Helmerhorst EJ Amerongen AV Antimicrobial peptides: Properties and applicability Biol. Chem 2001 382 597 619 11405223 10.1515/BC.2001.072

[22] Koczulla AR Bals R Antimicrobial peptides: Current status and therapeutic potential Drugs 2003 63 389 406 12558461 10.2165/00003495-200363040-00005

[23] Mookherjee N Brown KL Bowdish DM Doria S Falsafi R Hokamp K Roche FM Mu R Doho GH Pistolic J Powers JP Bryan J Brinkman FS Hancock RE Modulation of the TLR-mediated inflammatory response by the endogenous human host defense peptide LL-37 J. Immunol 2006 176 2455 2464 16456005 10.4049/jimmunol.176.4.2455

[24] Zanetti M The role of cathelicidins in the innate host defenses of mammals Curr. Issues Mol. Biol 2005 7 179 196 16053249

[25] Scott MG Davidson DJ Gold MR Bowdish D Hancock RE The human antimicrobial peptide LL-37 is a multifunctional modulator of innate immune responses J. Immunol 2002 169 3883 3891 12244186 10.4049/jimmunol.169.7.3883

[26] Koczulla R von Degenfeld G Kupatt C Krotz F Zahler S Gloe T Issbrucker K Unterberger P Zaiou M Lebherz C Karl A Raake P Pfosser A Boekstegers P Welsch U Hiemstra PS Vogelmeier C Gallo RL Clauss M Bals R An angiogenic role for the human peptide antibiotic LL-37/hCAP-18 J. Clin. Invest 2003 111 1665 1672 12782669 10.1172/JCI17545 PMC156109

[27] Jacobsen F Mittler D Hirsch T Gerhards A Lehnhardt M Voss B Steinau HU Steinstraesser L Transient cutaneous adenoviral gene therapy with human host defense peptide hCAP-18/LL-37 is effective for the treatment of burn wound infections Gene. Ther 2005 12 1494 1502 15973442 10.1038/sj.gt.3302568

[28] Shaykhiev R Beisswenger C Kandler K Senske J Puchner A Damm T Behr J Bals R Human endogenous antibiotic LL-37 stimulates airway epithelial cell proliferation and wound closure Am. J. Physiol. Lung Cell Mol. Physiol 2005 289 L842 L848 15964896 10.1152/ajplung.00286.2004

[29] Sieprawska-Lupa M Mydel P Krawczyk K Wojcik K Puklo M Lupa B Suder P Silberring J Reed M Pohl J Shafer W McAleese F Foster T Travis J Potempa J Degradation of human antimicrobial peptide LL-37 by Staphylococcus aureus-derived proteinases Antimicrob. Agents Chemother 2004 48 4673 4679 15561843 10.1128/AAC.48.12.4673-4679.2004 PMC529204

[30] Guina T Yi EC Wang H Hackett M Miller SI A PhoP-regulated outer membrane protease of Salmonella enterica serovar typhimurium promotes resistance to alpha-helical antimicrobial peptides J. Bacteriol 2000 182 4077 4086 10869088 10.1128/jb.182.14.4077-4086.2000 PMC94595

[31] Maemoto A Qu X Rosengren KJ Tanabe H Henschen-Edman A Craik DJ Ouellette AJ Functional analysis of the alpha-defensin disulfide array in mouse cryptdin-4 J. Biol. Chem 2004 279 44188 44196 15297466 10.1074/jbc.M406154200

[32] Campopiano DJ Clarke DJ Polfer NC Barran PE Langley RJ Govan JR Maxwell A Dorin JR Structure-activity relationships in defensin dimers: A novel link between beta-defensin tertiary structure and antimicrobial activity J. Biol. Chem 2004 279 48671 48679 15317821 10.1074/jbc.M404690200

[33] Wu Z Hoover DM Yang D Boulegue C Santamaria F Oppenheim JJ Lubkowski J Lu W Engineering disulfide bridges to dissect antimicrobial and chemotactic activities of human beta-defensin 3 Proc. Natl. Acad. Sci. USA 2003 100 8880 8885 12840147 10.1073/pnas.1533186100 PMC166407

[34] Yang D Biragyn A Hoover DM Lubkowski J Oppenheim JJ Beta-defensins: Linking innate and adaptive immunity through dendritic and T cell CCR6 Science 1999 286 525 528 10521347 10.1126/science.286.5439.525

[35] Gabay JE Scott RW Campanelli D Griffith J Wilde C Marra MN Seeger M Nathan CF Antibiotic proteins of human polymorphonuclear leukocytes Proc. Natl. Acad. Sci. USA 1989 86 5610 5614 2501794 10.1073/pnas.86.14.5610 PMC297672

[36] Lehrer RI Barton A Daher KA Harwig SS Ganz T Selsted ME Interaction of human defensins with Escherichia coli. mechanism of bactericidal activity J. Clin. Invest 1989 84 553 561 2668334 10.1172/JCI114198 PMC548915

[37] Panyutich AV Voitenok NN Lehrer RI Ganz T An enzyme immunoassay for human defensins J. Immunol. Methods 1991 141 149 155 1880422 10.1016/0022-1759(91)90141-2

[38] Panyutich AV Panyutich EA Krapivin VA Baturevich EA Ganz T Plasma defensin concentrations are elevated in patients with septicemia or bacterial meningitis J. Lab. Clin. Med 1993 122 202 207 8340706

[39] Panyutich A Ganz T Activated alpha 2-macroglobulin is a principal defensin-binding protein Am. J. Respir. Cell Mol. Biol 1991 5 101 106 1716445 10.1165/ajrcmb/5.2.101

[40] Panyutich AV Szold O Poon PH Tseng Y Ganz T Identification of defensin binding to C1 complement FEBS Lett 1994 356 169 173 7805831 10.1016/0014-5793(94)01261-x

[41] Tani K Murphy WJ Chertov O Salcedo R Koh CY Utsunomiya I Funakoshi S Asai O Herrmann SH Wang JM Kwak LW Oppenheim JJ Defensins act as potent adjuvants that promote cellular and humoral immune responses in mice to a lymphoma idiotype and carrier antigens Int. Immunol 2000 12 691 700 10784615 10.1093/intimm/12.5.691

[42] Lemaitre B Nicolas E Michaut L Reichhart JM Hoffmann JA The dorsoventral regulatory gene cassette spatzle/Toll/cactus controls the potent antifungal response in Drosophila adults Cell 1996 86 973 983 8808632 10.1016/s0092-8674(00)80172-5

[43] Chromek M Slamova Z Bergman P Kovacs L Podracka L Ehren I Hokfelt T Gudmundsson GH Gallo RL Agerberth B Brauner A The antimicrobial peptide cathelicidin protects the urinary tract against invasive bacterial infection Nat. Med 2006 12 636 641 16751768 10.1038/nm1407

[44] Thompson L Turko I Murad F Mass spectrometry-based relative quantification of human neutrophil peptides 1, 2, and 3 from biological samples Mol. Immunol 2006 43 1485 1489 16253330 10.1016/j.molimm.2005.08.001

[45] Zhou L Huang LQ Beuerman RW Grigg ME Li SF Chew FT Ang L Stern ME Tan D Proteomic analysis of human tears: Defensin expression after ocular surface surgery J. Proteome Res 2004 3 410 416 15253421 10.1021/pr034065n

[46] Hegedus CM Skibola CF Warner M Skibola DR Alexander D Lim S Dangleben NL Zhang L Clark M Pfeiffer RM Steinmaus C Smith AH Smith MT Moore LE Decreased urinary beta-defensin-1 expression as a biomarker of response to arsenic Toxicol. Sci 2008 106 74 82 18511430 10.1093/toxsci/kfn104 PMC2563143

[47] Coffelt SB Waterman RS Florez L Honer zu Bentrup K Zwezdaryk KJ Tomchuck SL LaMarca HL Danka ES Morris CA Scandurro AB Ovarian cancers overexpress the antimicrobial protein hCAP-18 and its derivative LL-37 increases ovarian cancer cell proliferation and invasion Int. J. Cancer 2008 122 1030 1039 17960624 10.1002/ijc.23186

[48] von Haussen J Koczulla R Shaykhiev R Herr C Pinkenburg O Reimer D Wiewrodt R Biesterfeld S Aigner A Czubayko F Bals R The host defence peptide LL-37/hCAP-18 is a growth factor for lung cancer cells Lung Cancer 2008 59 12 23 17764778 10.1016/j.lungcan.2007.07.014

[49] Bose SK Gibson W Bullard RS Donald CD PAX2 oncogene negatively regulates the expression of the host defense peptide human beta defensin-1 in prostate cancer Mol. Immunol 2009 46 1140 1148 19118900 10.1016/j.molimm.2008.11.004 PMC2680819

[50] Brown KL Hancock RE Cationic host defense (antimicrobial) peptides Curr. Opin. Immunol 2006 18 24 30 16337365 10.1016/j.coi.2005.11.004

[51] Boman HG Peptide antibiotics and their role in innate immunity Annu. Rev. Immunol 1995 13 61 92 7612236 10.1146/annurev.iy.13.040195.000425

[52] Yang D Biragyn A Kwak LW Oppenheim JJ Mammalian defensins in immunity: More than just microbicidal Trends Immunol 2002 23 291 296 12072367 10.1016/s1471-4906(02)02246-9

[53] Bals R Wilson JM Cathelicidins—A family of multifunctional antimicrobial peptides Cell. Mol. Life Sci 2003 60 711 720 12785718 10.1007/s00018-003-2186-9 PMC11138611

[54] Bowdish DM Davidson DJ Lau YE Lee K Scott MG Hancock RE Impact of LL-37 on anti-infective immunity J. Leukoc. Biol 2005 77 451 459 15569695 10.1189/jlb.0704380

[55] Morrison G Kilanowski F Davidson D Dorin J Characterization of the mouse beta defensin 1, Defb1, mutant mouse model Infect. Immun 2002 70 3053 3060 12010997 10.1128/IAI.70.6.3053-3060.2002 PMC128030

[56] Niyonsaba F Suzuki A Ushio H Nagaoka I Ogawa H Okumura K The human antimicrobial peptide dermcidin activates normal human keratinocytes Br. J. Dermatol 2009 160 243 249 19014393 10.1111/j.1365-2133.2008.08925.x

[57] Rieg S Steffen H Seeber S Humeny A Kalbacher H Dietz K Garbe C Schittek B Deficiency of dermcidin-derived antimicrobial peptides in sweat of patients with atopic dermatitis correlates with an impaired innate defense of human skin *in vivo* J. Immunol 2005 174 8003 8010 15944307 10.4049/jimmunol.174.12.8003

[58] Bals R Weiner DJ Moscioni AD Meegalla RL Wilson JM Augmentation of innate host defense by expression of a cathelicidin antimicrobial peptide Infect Immun 1999 67 6084 6089 10531270 10.1128/iai.67.11.6084-6089.1999 PMC96996

[59] Salzman NH Ghosh D Huttner KM Paterson Y Bevins CL Protection against enteric salmonellosis in transgenic mice expressing a human intestinal defensin Nature 2003 422 522 526 12660734 10.1038/nature01520

[60] Schittek B Paulmann M Senyurek I Steffen H The role of antimicrobial peptides in human skin and in skin infectious diseases Infect. Disord. Drug Targets 2008 8 135 143 18782030 10.2174/1871526510808030135

[61] Harder J Meyer-Hoffert U Wehkamp K Schwichtenberg L Schroder JM Differential gene induction of human beta-defensins (hBD-1, -2, -3, and -4) in keratinocytes is inhibited by retinoic acid J. Invest. Dermatol 2004 123 522 529 15304092 10.1111/j.0022-202X.2004.23234.x

[62] Proud D Sanders SP Wiehler S Human rhinovirus infection induces airway epithelial cell production of human beta-defensin 2 both *in vitro* and *in vivo* J. Immunol 2004 172 4637 4645 15034083 10.4049/jimmunol.172.7.4637

[63] Vora P Youdim A Thomas LS Fukata M Tesfay SY Lukasek K Michelsen KS Wada A Hirayama T Arditi M Abreu MT Beta-defensin-2 expression is regulated by TLR signaling in intestinal epithelial cells J. Immunol 2004 173 5398 5405 15494486 10.4049/jimmunol.173.9.5398

[64] Bhat S Milner S Antimicrobial peptides in burns and wounds Curr. Protein Pept. Sci 2007 8 506 520 17979765 10.2174/138920307782411428

[65] Vallender EJ Lahn BT Positive selection on the human genome Hum. Mol. Genet 2004 13 R245 R254 15358731 10.1093/hmg/ddh253

[66] Patil A Hughes AL Zhang G Rapid evolution and diversification of mammalian alpha-defensins as revealed by comparative analysis of rodent and primate genes Physiol. Genomics 2004 20 1 11 15494476 10.1152/physiolgenomics.00150.2004

[67] Andres E Dimarcq JL Cationic antimicrobial peptides: From innate immunity study to drug development. Update Med. Mal. Infect 2007 37 194 199 17306486 10.1016/j.medmal.2006.09.009

[68] Dorschner RA Lopez-Garcia B Peschel A Kraus D Morikawa K Nizet V Gallo RL The mammalian ionic environment dictates microbial susceptibility to antimicrobial defense peptides FASEB J 2006 20 35 42 16394265 10.1096/fj.05-4406com

[69] Johansson J Gudmundsson GH Rottenberg ME Berndt KD Agerberth B Conformation-dependent antibacterial activity of the naturally occurring human peptide LL-37 J. Biol. Chem 1998 273 3718 3724 9452503 10.1074/jbc.273.6.3718

[70] Oren Z Lerman JC Gudmundsson GH Agerberth B Shai Y Structure and organization of the human antimicrobial peptide LL-37 in phospholipid membranes: Relevance to the molecular basis for its non-cell-selective activity Biochem. J 1999 341 501 513 10417311 PMC1220385

[71] Hirsch T Jacobsen F Steinau HU Steinstraesser L Host defense peptides and the new line of defence against multiresistant infections Protein Pept. Lett 2008 15 238 248 18336350 10.2174/092986608783744252

[72] Larrick JW Hirata M Balint RF Lee J Zhong J Wright SC Human CAP18: A novel antimicrobial lipopolysaccharide-binding protein Infect. Immun 1995 63 1291 1297 7890387 10.1128/iai.63.4.1291-1297.1995 PMC173149

[73] Steinstraesser L Tippler B Mertens J Lamme E Homann HH Lehnhardt M Wildner O Steinau HU Uberla K Inhibition of early steps in the lentiviral replication cycle by cathelicidin host defense peptides Retrovirology 2005 2 2 14 15656908 10.1186/1742-4690-2-2 PMC548510

[74] Zasloff M Antimicrobial peptides of multicellular organisms Nature 2002 415 389 395 11807545 10.1038/415389a

[75] Bowdish DM Hancock RE Anti-endotoxin properties of cationic host defence peptides and proteins J. Endotoxin Res 2005 11 230 236 16176660 10.1179/096805105X37394

[76] Hancock RE Rozek A Role of membranes in the activities of antimicrobial cationic peptides FEMS Microbiol. Lett 2002 206 143 149 11814654 10.1111/j.1574-6968.2002.tb11000.x

[77] Hale JD Hancock RE Alternative mechanisms of action of cationic antimicrobial peptides on bacteria Expert Rev. Anti. Infect. Ther 2007 5 951 959 18039080 10.1586/14787210.5.6.951

[78] Lohner K Blondelle SE Molecular mechanisms of membrane perturbation by antimicrobial peptides and the use of biophysical studies in the design of novel peptide antibiotics Comb. Chem. High Throughput Screen 2005 8 241 256 15892626 10.2174/1386207053764576

[79] Yang D Biragyn A Hoover DM Lubkowski J Oppenheim JJ Multiple roles of antimicrobial defensins, cathelicidins, and eosinophil-derived neurotoxin in host defense Annu. Rev. Immunol 2004 22 181 215 15032578 10.1146/annurev.immunol.22.012703.104603

[80] Finlay BB Hancock RE Can innate immunity be enhanced to treat microbial infections? Nat. Rev. Microbiol 2004 2 497 504 15152205 10.1038/nrmicro908

[81] Hiemstra PS Fernie-King BA McMichael J Lachmann PJ Sallenave JM Antimicrobial peptides: Mediators of innate immunity as templates for the development of novel anti-infective and immune therapeutics Curr. Pharm. Des 2004 10 2891 2905 15379675 10.2174/1381612043383566

[82] Selsted ME Ouellette AJ Mammalian defensins in the antimicrobial immune response Nat. Immunol 2005 6 551 557 15908936 10.1038/ni1206

[83] Braff MH Hawkins MA Di Nardo A Lopez-Garcia B Howell MD Wong C Lin K Streib JE Dorschner R Leung DY Gallo RL Structure-function relationships among human cathelicidin peptides: Dissociation of antimicrobial properties from host immunostimulatory activities J. Immunol 2005 174 4271 4278 15778390 10.4049/jimmunol.174.7.4271

[84] Davidson DJ Currie AJ Reid GS Bowdish DM MacDonald KL Ma RC Hancock RE Speert DP The cationic antimicrobial peptide LL-37 modulates dendritic cell differentiation and dendritic cell-induced T cell polarization J. Immunol 2004 172 1146 1156 14707090 10.4049/jimmunol.172.2.1146

[85] Nagaoka I Hirota S Niyonsaba F Hirata M Adachi Y Tamura H Heumann D Cathelicidin family of antibacterial peptides CAP18 and CAP11 inhibit the expression of TNF-alpha by blocking the binding of LPS to CD14(+) cells J. Immunol 2001 167 3329 3338 11544322 10.4049/jimmunol.167.6.3329

[86] Befus AD Mowat C Gilchrist M Hu J Solomon S Bateman A Neutrophil defensins induce histamine secretion from mast cells: Mechanisms of action J. Immunol 1999 163 947 953 10395691

[87] Niyonsaba F Iwabuchi K Someya A Hirata M Matsuda H Ogawa H Nagaoka I A cathelicidin family of human antibacterial peptide LL-37 induces mast cell chemotaxis Immunology 2002 106 20 26 11972628 10.1046/j.1365-2567.2002.01398.x PMC1782699

[88] Salzet M Antimicrobial peptides are signaling molecules Trends Immunol 2002 23 283 284 12072365 10.1016/s1471-4906(02)02236-6

[89] Remick DG Pathophysiology of sepsis Am. J. Pathol 2007 170 1435 1444 17456750 10.2353/ajpath.2007.060872 PMC1854939

[90] Russell JA Management of sepsis N. Engl. J. Med 2006 355 1699 1713 17050894 10.1056/NEJMra043632

[91] Calandra T Bochud PY Heumann D Cytokines in septic shock Curr. Clin. Top. Infect. Dis 2002 22 1 23 12520644

[92] Byl B Clevenbergh P Kentos A Jacobs F Marchant A Vincent JL Thys JP Ceftazidime- and imipenem-induced endotoxin release during treatment of gram-negative infections Eur. J. Clin. Microbiol. Infect. Dis 2001 20 804 807 11783697 10.1007/s100960100609

[93] Parker SJ Watkins PE Experimental models of gram-negative sepsis Br. J. Surg 2001 88 22 30 11136305 10.1046/j.1365-2168.2001.01632.x

[94] Shenep JL Barton RP Mogan KA Role of antibiotic class in the rate of liberation of endotoxin during therapy for experimental gram-negative bacterial sepsis J. Infect. Dis 1985 151 1012 1018 3889171 10.1093/infdis/151.6.1012

[95] Scott MG Vreugdenhil AC Buurman WA Hancock RE Gold MR Cutting edge: Cationic antimicrobial peptides block the binding of lipopolysaccharide (LPS) to LPS binding protein J. Immunol 2000 164 549 553 10623792 10.4049/jimmunol.164.2.549

[96] Cirioni O Giacometti A Ghiselli R Bergnach C Orlando F Silvestri C Mocchegiani F Licci A Skerlavaj B Rocchi M Saba V Zanetti M Scalise G LL-37 protects rats against lethal sepsis caused by gram-negative bacteria Antimicrob. Agents Chemother 2006 50 1672 1679 16641434 10.1128/AAC.50.5.1672-1679.2006 PMC1472226

[97] McPhee JB Hancock RE Function and therapeutic potential of host defence peptides J. Pept. Sci 2005 11 677 687 16103989 10.1002/psc.704

[98] Murphy CJ Foster BA Mannis MJ Selsted ME Reid TW Defensins are mitogenic for epithelial cells and fibroblasts J. Cell Physiol 1993 155 408 413 8482733 10.1002/jcp.1041550223

[99] Chavakis T Cines DB Rhee JS Liang OD Schubert U Hammes HP Higazi AA Nawroth PP Preissner KT Bdeir K Regulation of neovascularization by human neutrophil peptides (alpha-defensins): A link between inflammation and angiogenesis FASEB J 2004 18 1306 1308 15208269 10.1096/fj.03-1009fje

[100] Steinstraesser L Ring A Bals R Steinau HU Langer S The human host defense peptide LL37/hCAP accelerates angiogenesis in PEGT/PBT biopolymers Ann. Plast. Surg 2006 56 93 98 16374104 10.1097/01.sap.0000190883.30005.91

[101] Sorensen OE Cowland JB Theilgaard-Monch K Liu L Ganz T Borregaard N Wound healing and expression of antimicrobial peptides/polypeptides in human keratinocytes, a consequence of common growth factors J. Immunol 2003 170 5583 5589 12759437 10.4049/jimmunol.170.11.5583

[102] Heilborn JD Nilsson MF Kratz G Weber G Sorensen O Borregaard N Stahle-Backdahl M The cathelicidin anti-microbial peptide LL-37 is involved in re-epithelialization of human skin wounds and is lacking in chronic ulcer epithelium J. Invest. Dermatol 2003 120 379 389 12603850 10.1046/j.1523-1747.2003.12069.x

[103] Yount NY Bayer AS Xiong YQ Yeaman MR Advances in antimicrobial peptide immunobiology Biopolymers 2006 84 435 458 16736494 10.1002/bip.20543

[104] Peschel A How do bacteria resist human antimicrobial peptides? Trends Microbiol 2002 10 179 186 11912025 10.1016/s0966-842x(02)02333-8

[105] Jin T Bokarewa M Foster T Mitchell J Higgins J Tarkowski A *Staphylococcus aureus* resists human defensins by production of staphylokinase, a novel bacterial evasion mechanism J. Immunol 2004 172 1169 1176 14707093 10.4049/jimmunol.172.2.1169

[106] Frick IM Akesson P Rasmussen M Schmidtchen A Bjorck L SIC, a secreted protein of Streptococcus pyogenes that inactivates antibacterial peptides J. Biol. Chem 2003 278 16561 16566 12621031 10.1074/jbc.M301995200

[107] Shafer WM Qu XD Waring AJ Lehrer RI Modulation of *Neisseria gonorrhoeae* susceptibility to vertebrate antibacterial peptides due to a member of the resistance/nodulation/division efflux pump family Proc. Natl. Acad. Sci. USA 1998 95 1829 1833 9465102 10.1073/pnas.95.4.1829 PMC19198

[108] Tzeng YL Ambrose KD Zughaier S Zhou X Miller YK Shafer WM Stephens DS Cationic antimicrobial peptide resistance in *Neisseria meningitidis* J. Bacteriol 2005 187 5387 5396 16030233 10.1128/JB.187.15.5387-5396.2005 PMC1196002

[109] Hancock RE Sahl HG Antimicrobial and host-defense peptides as new anti-infective therapeutic strategies Nat. Biotechnol 2006 24 1551 1557 17160061 10.1038/nbt1267

[110] Cox CR Coburn PS Gilmore MS Enterococcal cytolysin: A novel two component peptide system that serves as a bacterial defense against eukaryotic and prokaryotic cells Curr. Protein Pept. Sci 2005 6 77 84 15638770 10.2174/1389203053027557

[111] Scott MG Hancock RE Cationic antimicrobial peptides and their multifunctional role in the immune system Crit. Rev. Immunol 2000 20 407 431 11145218

[112] Ganz T Selsted ME Lehrer RI Defensins Eur. J. Haematol 1990 44 1 8 2407547 10.1111/j.1600-0609.1990.tb00339.x

[113] Shai Y Mechanism of the binding, insertion and destabilization of phospholipid bilayer membranes by alpha-helical antimicrobial and cell non-selective membrane-lytic peptides Biochim. Biophys. Acta 1999 1462 55 70 10590302 10.1016/s0005-2736(99)00200-x

[114] Matsuzaki K Why and how are peptide-lipid interactions utilized for self-defense? Magainins and tachyplesins as archetypes Biochim. Biophys. Acta 1999 1462 1 10 10590299 10.1016/s0005-2736(99)00197-2

[115] Yang L Weiss TM Lehrer RI Huang HW Crystallization of antimicrobial pores in membranes: Magainin and protegrin Biophys. J 2000 79 2002 2009 11023904 10.1016/S0006-3495(00)76448-4 PMC1301090

[116] Dathe M Meyer J Beyermann M Maul B Hoischen C Bienert M General aspects of peptide selectivity towards lipid bilayers and cell membranes studied by variation of the structural parameters of amphipathic helical model peptides Biochim. Biophys. Acta 2002 1558 171 186 11779567 10.1016/s0005-2736(01)00429-1

[117] Fukumoto K Nagaoka I Yamataka A Kobayashi H Yanai T Kato Y Miyano T Effect of antibacterial cathelicidin peptide CAP18/LL-37 on sepsis in neonatal rats Pediatr. Surg. Int 2005 21 20 24 15645239 10.1007/s00383-004-1256-x

[118] Torossian A Gurschi E Bals R Vassiliou T Wulf HF Bauhofer A Effects of the antimicrobial peptide LL-37 and hyperthermic preconditioning in septic rats Anesthesiology 2007 107 437 441 17721246 10.1097/01.anes.0000278906.86815.eb

[119] Oren Z Lerman JC Gudmundsson GH Agerberth B Shai Y Structure and organization of the human antimicrobial peptide LL-37 in phospholipid membranes: Relevance to the molecular basis for its non-cell-selective activity Biochem. J 1999 341 501 513 10417311 PMC1220385

[120] Ciornei CD Egesten A Bodelsson M Effects of human cathelicidin antimicrobial peptide LL-37 on lipopolysaccharide-induced nitric oxide release from rat aorta *in vitro* Acta Anaesthesiol. Scand 2003 47 213 220 12631052 10.1034/j.1399-6576.2003.00045.x

[121] Niyonsaba F Ushio H Nagaoka I Okumura K Ogawa H The human beta-defensins (-1, -2, -3, -4) and cathelicidin LL-37 induce IL-18 secretion through p38 and ERK MAPK activation in primary human keratinocytes J. Immunol 2005 175 1776 1784 16034119 10.4049/jimmunol.175.3.1776

[122] Scott MG Davidson DJ Gold MR Bowdish D Hancock RE The human antimicrobial peptide LL-37 is a multifunctional modulator of innate immune responses J. Immunol 2002 169 3883 3891 12244186 10.4049/jimmunol.169.7.3883

[123] Steinstraesser L Koehler T Jacobsen F Daigeler A Goertz O Langer S Kesting M Steinau H Eriksson E Hirsch T Host defense peptides in wound healing Mol. Med 2008 14 528 537 18385817 10.2119/2008-00002.Steinstraesser PMC2277318

[124] Kaus A Jacobsen F Sorkin M Rittig A Voss B Daigeler A Sudhoff H Steinau HU Steinstraesser L Host defence peptides in human burns Burns 2008 34 32 40 17714876 10.1016/j.burns.2007.04.017

[125] Jacobsen F Mohammadi-Tabrisi A Hirsch T Mittler D Mygind PH Sonksen CP Raventos D Kristensen HH Gatermann S Lehnhardt M Daigeler A Steinau HU Steinstraesser L Antimicrobial activity of the recombinant designer host defence peptide P-novispirin G10 in infected full-thickness wounds of porcine skin J. Antimicrob. Chemother 2007 59 493 498 17289767 10.1093/jac/dkl513

[126] Hirsch T von Peter S Dubin G Mittler D Jacobsen F Lehnhardt M Eriksson E Steinau HU Steinstraesser L Adenoviral gene delivery to primary human cutaneous cells and burn wounds Mol. Med 2006 12 199 207 17225867 10.2119/2006-00031.Hirsch PMC1770006

[127] Steinstraesser L Tack BF Waring AJ Hong T Boo LM Fan MH Remick DI Su GL Lehrer RI Wang SC Activity of novispirin G10 against Pseudomonas aeruginosa *in vitro* and in infected burns Antimicrob. Agents. Chemother 2002 46 1837 1844 12019098 10.1128/AAC.46.6.1837-1844.2002 PMC127209

[128] Sigurdardottir T Andersson P Davoudi M Malmsten M Schmidtchen A Bodelsson M In silico identification and biological evaluation of antimicrobial peptides based on human cathelicidin LL-37 Antimicrob. Agents Chemother 2006 50 2983 2989 16940092 10.1128/AAC.01583-05 PMC1563516

[129] Eckert R Qi F Yarbrough DK He J Anderson MH Shi W Adding selectivity to antimicrobial peptides: Rational design of a multidomain peptide against Pseudomonas spp Antimicrob. Agents Chemother 2006 50 1480 1488 16569868 10.1128/AAC.50.4.1480-1488.2006 PMC1426969

[130] Eckert R Greenberg EP Qi F Yarbrough DK He J McHardy I Anderson MH Shi W Enhancement of antimicrobial activity against pseudomonas aeruginosa by coadministration of G10KHc and tobramycin Antimicrob. Agents Chemother 2006 50 3833 3838 16940063 10.1128/AAC.00509-06 PMC1635211

[131] Kristian SA Timmer AM Liu GY Lauth X Sal-Man N Rosenfeld Y Shai Y Gallo RL Nizet V Impairment of innate immune killing mechanisms by bacteriostatic antibiotics FASEB J 2007 21 1107 1116 17215486 10.1096/fj.06-6802com

[132] Ghiselli R Giacometti A Cirioni O Mocchegiani F Orlando F D’Amato G Sisti V Scalise G Saba V Cecropin B enhances betalactams activities in experimental rat models of gram-negative septic shock Ann. Surg 2004 239 251 256 14745334 10.1097/01.sla.0000108673.25385.03 PMC1356219

[133] Zhang L Falla TY Antimicrobial peptides: Therapeutic potential Expert. Opin. Pharmacother 2006 7 653 663 16556083 10.1517/14656566.7.6.653

[134] Levin M Quint PA Goldstein B Barton P Bradley JS Shemie SD Yeh T Kim SS Cafaro DP Scannon PJ Giroir BP Recombinant bactericidal/permeability-increasing protein (rBPI21) as adjunctive treatment for children with severe meningococcal sepsis: A randomised trial. rBPI21 Meningococcal Sepsis Study Group Lancet 2000 356 961 967 11041396 10.1016/s0140-6736(00)02712-4

[135] Hancock RE Patrzykat A Clinical development of cationic antimicrobial peptides: From natural to novel antibiotics Curr. Drug Targets Infect. Disord 2002 2 79 83 12462155 10.2174/1568005024605855

